# *In vitro* Effect of Recombinant Feline Interferon-Ω (rFeIFN-Ω) on the Primary CanineTransmissible Venereal Tumor Culture

**DOI:** 10.3389/fvets.2019.00104

**Published:** 2019-04-09

**Authors:** Chanokchon Setthawongsin, Sirikachorn Tangkawattana, Anudep Rungsipipat, Somporn Techangamsuwan

**Affiliations:** ^1^Companion Animal Cancer Research Unit, Department of Pathology, Faculty of Veterinary Science, Chulalongkorn University, Bangkok, Thailand; ^2^Department of Veterinary Pathobiology, Faculty of Veterinary Medicine, KhonKaen University, KhonKaen, Thailand

**Keywords:** CTVT, interferon, *in vitro* study, primary culture, rFeIFN-Ω

## Abstract

**Background:** Interferons (IFNs), signaling proteins produced by host cells, are secreted in response to pathogen activity as well as to tumor cells, and display antiviral, antiproliferative, and immunomodulatory effects. Recombinant feline interferon omega (rFeIFN-ω) has *in vitro* growth inhibition activities on various canine and feline tumor cell lines. Canine transmissible venereal tumor (CTVT) is used as an animal model for immunotherapy due to its specific growth phase. Previous studies have usually focused on the interaction between tumor infiltrating lymphocytes (TILs) and CTVT cells. However, the specific effects of rFeIFN-ω on CTVT cells remains poorly defined.

**Aims:** The aims of this study, therefore, were to evaluate the *in vitro* effect of rFeIFN-ω on primary CTVT cells and to study the mRNA expression of apoptotic genes and drug resistance genes.

**Materials and Methods:** Purified CTVT cells were treated with various concentrations of rFeIFN-ω and the viability of the cultured cells was ascertained at 24, 48, and 72 h post treatment (hpt) and a dose-response curve plotted. The mRNA expression of apoptotic (*BAX* and *BCL-2*) and drug resistance (*ABCB1* and *ABCG2*) genes was performed by reverse transcription quantitative real-time PCR at 72 hpt.

**Results:** rFeIFN-ω displayed an effect against CTVT cell viability, which decreasing viability in a dose-dependent manner within 72 hpt. The relative mRNA expression of *BCL-2* was upregulated only at a rFeIFN-ω concentration of 10^4^ IU/100 μl. However, higher concentrations of rFeIFN-ω gave a higher level of relative mRNA expression of *ABCB1* transporter gene.

**Conclusion:** This study provided the information of *in vitro* effect of rFeIFN-ω on CTVT cell viability in a dose dependent manner, as well as, the alteration of *BCL-2* and *ABCB1* gene expression after treatment. These results encourage future *in vivo* studies to evaluate the potential efficacy of this treatment in CTVT cases.

## Introduction

Interferons (IFNs) are signaling proteins (cytokines) produced by host cells and play a role in immunological responses to various stimuli or inducers, such as viruses. Besides their antiviral activity, IFNs also have an antiproliferative capacity, anti-inflammatory potential and immunomodulatory effects. Basically, IFNs are classified into three types. The type I IFNs (IFN-Is), or viral interferons, which include IFN-α (leukocyte IFN), IFN-β (fibroblast IFN), IFN-δ, -τ, and -ω. The IFN-Is have the ability to inhibit tumor cell growth and induce apoptosis, activate Natural killer (NK) cell activity and increase the expression of MHC class I molecules. The type II IFN (IFN-II) or immune interferon, comprised of only IFN-γ, increases the expression of MHC on antigen presenting cells, modulates B-lymphocyte responses and activates NK cells. Lastly, the type III IFN (IFN-III) or IFN-λ, is synthesized only by epithelial cells. Overall, IFNs are involved in both the innate and adaptive immunity, not only in their regulation but also in the activation of the immune response (1, 2).

Initially, IFNs were used for treatment based on the non-specific inducers of type I IFNs, and then subsequently replaced by the recombinant proteins. The first recombinant human type I IFN was produced as protein from *Escherichia coli* in the 1980s (3). Then, recombinant canine IFNs were constructed and used for treating viral diseases (4–6). Clinical application of IFN-Is in animal oncology has included the treatment of feline fibrosarcoma and *in vitro* research in nasal adenocarcinoma, thyroid carcinoma, squamous cell carcinoma, lymphoma, osteosarcoma, and melanoma (7, 8).

For *in vitro* studies, canine and feline cell lines derived from round cell tumors showed a higher sensitivity to IFNs and cobalt-60 radiation than those from solid tumors. For IFN-ω, it was first discovered in human in 1985 (3) and later in horse, pig, rabbit, cattle and cat (9). Interestingly, several reports have stated that IFN-ω genes were derived in the canine genome by modified deletion of some genes (4–6). However, IFN- ω still was not found in canine and mice. It was suggested that all INF-ω protein evolved from a common ancestor based on phylogenetic tree analysis (9). Previous studies have suggested that IFN-ω has antiproliferative effects, where recombinant feline (rFe) IFN-ω showed *in vitro* growth inhibition of various canine and feline tumor cell lines (10–12).

Canine transmissible venereal tumor (CTVT) is a natural contagious tumor in domestic dogs (*Canis familiaris*). Experimental CTVT transplantation was performed in healthy and immunosuppressed dogs and other social canids, such as wolf (*Canis lupus*), coyote (*Canis latrans*), fox (genus Vulpes) and jackal (*Canis aureus*), and was xenografted in SCID mice (13, 14). After transmission or transplantation, the tumor mass remains localized to the site of inoculation. The ancestor of CTVT cells is believed to be associated with wolves or an East Asian dog breed with a low major histocompatibility complex (MHC) diversity. The transplantation ability of live CTVT cells is restricted by the relationship between the host and CTVT co-evolution. The CTVT cells evade the host immune system by down-regulation of their cell surface expression of MHC class I and II, allowing their transmission by natural allografts (13, 15).

In addition, CTVT possesses an interesting growth pattern which not only occurs in natural transmission but also in experimental transplantation (14). In the progression phase, CTVT cells escape host rejection by suppressing their MHC expression levels, decreasing NK cell activity, and impairing the function of host T cell and dendritic cells. These are affected by CTVT-released transforming growth factor-β (TGF-β), which modulates their MHC expression level and decreases the function of the host IFN-γ. Interestingly, CTVT has a unique regression feature which spontaneously occurs due to the immune reaction between CTVT and tumor infiltrating lymphocytes (TILs). During the regression phase, the host interleukin-6 (IL-6) from TILs and host IFN-γ act concomitantly to enhance the expression of MHC molecules (16–18).

In general, IFNs affect cell proliferation, immune response, angiogenesis inhibition, and apoptosis promotion (19–21). The Bcl-family, consisting of both pro- and anti-apoptotic proteins, is involved in programmed cell death, or apoptosis, by the intrinsic or mitochondrial pathway. The balance between pro-apoptotic (Bax) and anti-apoptotic (Bcl-2) proteins is the important prognostic criteria. Overexpression of Bcl-2 is related to the malignant behavior and drug resistance phenomena of cancer, since Bcl-2 prevents apoptosis and supports cell viability without promoting cell proliferation, by inhibiting the activity of Bax (22–25).

The drug transporters are membrane proteins that have the primary function to facilitate the flux of molecules into and out of cells. P-glycoprotein (P-gp), or ABCB1, was the first well-known member of the ATP-binding cassette (ABC) group of transporters. In addition, the *ABCC1* (MRP1), *ABCC2* (MRP2), and *ABCG2* (BCRP) genes (proteins) are also included. Overexpression of P-gp is related to the multidrug resistance (MDR) phenomena (26–28), where tumor cells are selected for resistance to a given drug and the resistance encompasses other compounds that are structurally or functionally unrelated. Previous *in vitro* studies reported higher expression levels of *MDR-1* in CTVT cells that showed a prolonged survival rate after vincristine sulfate application. Moreover, the plasmacytoid cell type CTVT expressed a high P-gp expression level with a potential drug resistance (29, 30).

Overall, CTVT is considered as an animal-derived tumor model for immunotherapeutic studies due to its specific growth phase. Previous studies have usually focused on the interaction between TILs and CTVT cells, but the information on the effect of IFNs on CTVT cells is limited. Accordingly, the aims of this study were to evaluate the *in vitro* effect of recombinant feline interferon omega (rFeIFN-ω) on a primary culture of CTVT cells and to study the mRNA expression of apoptotic genes and drug resistance genes after treated with rFeIFN-ω.

## Materials and Methods

### Animal and Sample Collection

The genital CTVT mass of a 4-year-old mongrel male dog was diagnosed by cytological and polymerase chain reaction (PCR) examination (Supplementary Figure 1), as reported (31). This dog was intravascularly injected with tiletamine-zolazepam (Zoletil®, Virbac, France) at a dosage of 4 mg/kg body weight (BW). The tumor tissue was aseptically biopsied before commencing chemotherapeutic treatment on the dog, as weekly intravenous injection of vincristine sulfate at 0.025 mg/kg BW. Sampling procedures were approved by the Chulalongkorn University Animal Care and Use Committee (No. 133100077). Biopsied tissue was immediately immersed in Hank's buffered salt solution (HBSS; Gibco Lab, USA) containing 100 IU/ml penicillin and 100 μg/ml streptomycin (Gibco Laboratories, USA). The dog's hematology and serum chemistry profile were in the normal range prior to receiving chemotherapeutic treatment.

### Primary Culture of CTVT Cells Deprived of TILs

The sterile CTVT mass (~1.5 cm^3^) was minced in HBSS and crushed using cell strainer (SPL Life Sciences, Korea) to obtain a uniform single cell suspension. The suspension was then overlaid on a gradient of 42% Percoll (GE Healthcare, Sigma-Aldrich, USA) and centrifuged at 820 × g, 4°C, for 25 min. The thin layer of (suspected CTVT) cells at the Percoll-HBSS interface were harvested and washed three times with Dulbecco's Modified Eagle Medium (DMEM; Gibco Lab, USA) by centrifugation (820x g, 4°C, for 25 min) (16, 30). The washed CTVT cells were resuspended, counted and seeded in a 24-well plate at 2.78 × 10^6^ cells/well in DMEM media supplemented with 10% (v/v) fetal bovine serum (FBS; Gibco Lab, USA) and incubated at 37°C in 5% (v/v) CO_2_.

### Effect of rFeIFN-ω on the Viability of CTVT Cells

Serial dilutions of rFeIFN-ω (Virbac, France) were prepared at 10^3^, 10^4^, 10^5^, 10^6^, and 2 × 10^6^ IU/100 μl, and added to each well soon after CTVT cell isolation (day 0 or 0 h). Cell viability was evaluated by trypan blue dye exclusion, and cell counting using a hematocytometer. The results are reported as the total number of viable cells/well at 0, 24, 48, and 72 h post treatment (hpt) with rFeIFN-ω. The results from triplicate wells were averaged and presented as the mean percentage viability of treated CTVT cells compared with their control counterparts (untreated CTVT cells) at the same time point. Means and standard error of mean (SEM) of the percentage of the viability of control cells were plotted with varied rFeIFN-ω concentrations to generate a dose-response curve.

### mRNA Expression of Apoptotic and Drug Resistance Genes

Total RNA was extracted from the endpoint of the primary CTVT culture after IFN treatment (72 hpt) using a Nucleospin RNA kit (Macherey-Nagel, Germany) according to the manufacturer's protocol. Briefly, the CTVT cell suspension was lysed, and the total lysate was trapped in the membrane column. The genomic DNA was removed by treating the samples with RQ1 RNase-Free DNase (Promega, Madison, USA), and then the total RNA was eluted. The RNA concentration and purity were determined using a NanoDrop Lite spectrophotometer (Thermo Fisher Scientific Inc., Wilmington, USA).

For the two-step reverse transcription quantitative real-time PCR (RT-qPCR), in the first step, 25 ng of total RNA was used as the template to construct cDNA using an Omniscript® Reverse Transcription Kit (Qiagen, Germany). The second step qPCR was performed using Rotor gene® Q thermocycler (Qiagen, Germany) with KAPA SYBR® Fast qPCR Kit Master Mix (2X) universal, and the respective oligonucleotide primer pair for the housekeeping gene (β*-ACTIN*) or the genes of interest (*BAX, BCL-2, ABCB1*, and *ABCG2*), shown in Table 1 (32–34).

**Table 1:** Primers used for real time PCR assays.

**Gene**	**Sequence**	**Product (bp)**	**References**
*β-ACTIN*	5′-ATG GAA TCA TGC GGT ATC CAC-3′ 5′-CTT CTG CAT CCT GTC AGC AA-3′	141	(32)
*ABCB1*	5′-CAG TGG TTC AGG TGG CCC T-3′ 5′-CGA ACT GTA GAC AAA CGA TGA GCT-3′	79	(33)
*ABCG2*	5′-GGT ATC CAT AGC AAC TCT CCT CA-3′ 5′-GCA AAG CCG CAT AAC CAT-3′	143	(33)
*BCL-2*	5′-CAT GCC AAG AGG GAA ACA CCA GAA-3′ 5′-GTG CTT TGC ATT CTT GGA TGA GGG-3′	76	(34)
*BAX*	5′-TTC CGA GTG GCA GCT GAG ATG TTT-3′ 5′-TGC TGG CAA AGT AGA AGA GGG CAA-3′	79	(34)

Briefly, each qPCR reaction was comprised of 10 μl of 2X qPCR Master Mix, 200 nM of each primer and 1.25 ng of cDNA and adjusted to a final volume of 20 μl. The reaction was thermocycled at 95°C for 3 min, followed by 40 cycles of 95°C for 3 s and 60°C for 30 s. The threshold of fluorescence detection was set at the number of the threshold cycles (Ct) corresponding to the inflection point of the fluorescence curve from the baseline to the exponential phase. Each reaction was performed in duplicate intra-assay and inter-assay validations. A melting curve ranging from 75 to 95°C was set to analyze the specificity of the PCR product. The real-time PCR efficiency ranged from 85 to 100%, which was derived from the standard curves of a normal dog cDNA dilution series. A no template control was included in each analysis. For each treated sample, the Ct value where the duplicate Ct values differed by <1 was used for analysis of the relative gene expression with the control group by the 2^−ΔΔct^ method.

### Statistical Analysis

A one-way analysis of variance (ANOVA) was used to compare the means, followed by the Tukey's Multiple Comparison test, where *P* < 0.05 was considered as significant. The analysis was performed with GraphPad Prism program (GraphPad Software, USA).

## Results

### Animal Treatment Response

The CTVT-bearing dog was treated with six administrations of vincristine sulfate over 8 weeks and then neutered 1 month after complete remission. The adverse effect of vincristine sulfate was anorexia, weight loss, lethargy, and leucopenia, so the treatment was postponed in weeks 1 and 4.

### Viability of CTVT Cells

**Figure 1:**
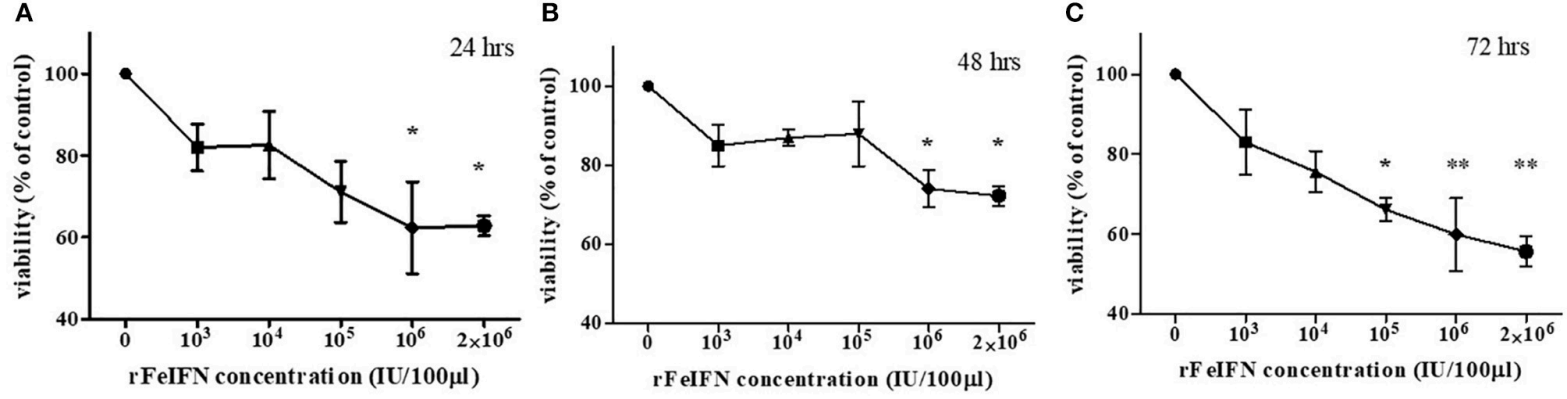
A dose responsive curve between rFeIFN-ω and cell viability at 24 **(A)**, 48 **(B)**, 72 **(C)** hours after treatment. (^*^p < 0.05; ^**^p < 0.01).

The viability of the primary CTVT culture treated with various concentrations of rFeIFN-ω at 24, 48, and 72 hpt were evaluated, and are shown in Figure 1. At 24 hpt, the viability of the CTVT cells had slightly decreased when treated with higher concentrations of rFeIFN-ω, with a viability of CTVT cells of 81.9, 82.6, 71.1, 63.3, and 65.5% at rFeIFN-ω concentrations of 10^3^, 10^4^, 10^5^, 10^6^, and 2 × 10^6^ IU/100 μl, respectively, when compared with the control cells (100%), but was only significantly different for the higher rFeIFN-ω concentrations of 10^6^ and 2 × 10^6^ IU/100 μl (Figure 1A). Similar findings were also observed at 48 hpt, where the mean viability of CTVT cells were 85.0, 87.0, 87.9, 74.1, and 72.2% at rFeIFN-ω concentrations of 10^3^, 10^4^, 10^5^, 10^6^, and 2 × 10^6^ IU/100 μl, respectively (Figure 1B). At 72 hpt, a significant decrease in the CTVT cell viability was seen with a rFeIFN-ω concentration of 10^5^ IU/100 μl, reflecting the greater sensitivity of CTVT cells to rFeIFN-ω compared with the 24- and 48-hpt cultures. The mean percentage of CTVT cell viability at 72 hpt were 83.0, 75.6, 66.1, 59.9, and 55.7% at 10^3^, 10^4^, 10^5^, 10^6^, and 2 × 10^6^ IU/100 μl, respectively (Figure 1C).

### The Relative mRNA Expression of the Apoptotic Genes (*BCL-2* and *BAX*) in rFeIFN-ω Treated CTVT Cultures

**Figure 2:**
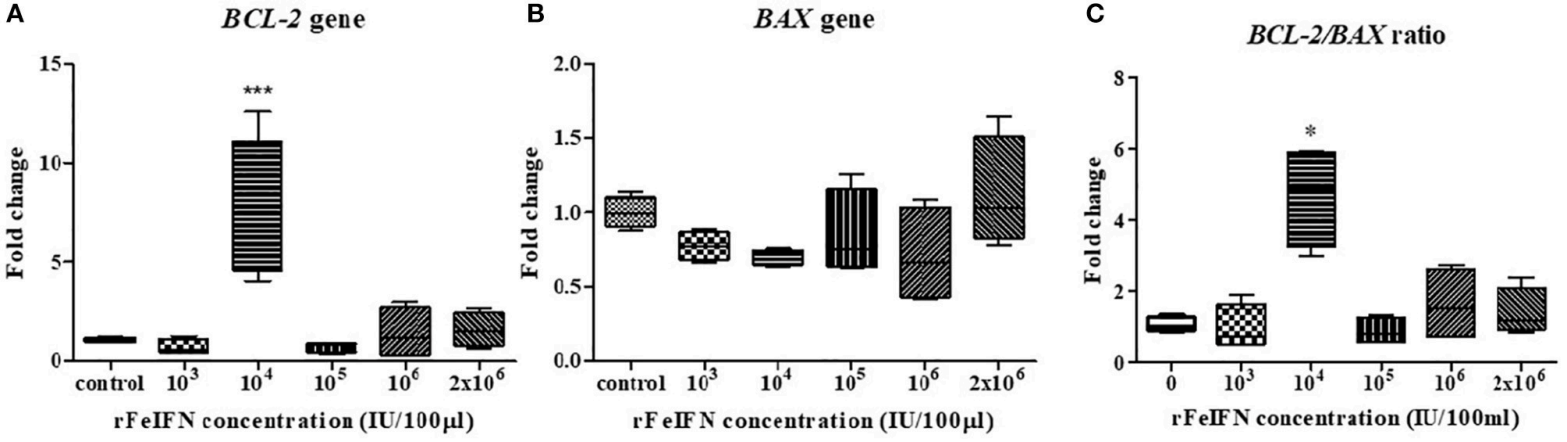
Box plot showed the relative mRNA expression of *BCL-2***(A)**, *BAX* **(B)** gene, and the relative *BCL-2*/*BAX* ratio **(C)** of primary CTVT culture at 72-h after incubation with varied concentrations of rFeIFN-ω (^*^p < 0.05; ^***^p < 0.001).

The relative mRNA expression level of *BCL-2* and *BAX* genes at the endpoint of the study (72 hpt) was quantitatively measured, normalized against β*-ACTIN* and compared with its control (at 0 hpt), with the results shown in Figure 2. The average relative mRNA expression level of *BCL-2* at 72 hpt was lower than in the control at rFeIFN-ω concentrations of 10^3^ and 10^5^ IU/100 μl (0.70- and 0.71-fold, respectively), but the *BCL-2* expression level was higher at rFeIFN-ω concentrations of 10^6^ and 2 × 10^6^ IU/100 μl (1.40- and 1.58-fold, respectively). However, the average relative transcriptional level was significantly and markedly (3.26-fold) higher than in the control at a rFeIFN-ω concentration of 10^4^ IU/100 μl (Figure 2A).

The average transcriptional level of *BAX* in the 72-hpt treated culture was not significantly different from that in the control, but showed numerically lower expressions at almost all concentrations of rFeIFN-ω compared to the control at 0.77-, 0.69-, 0.84- and 0.71-fold lower at 10^3^, 10^4^, 10^5^, and 10^6^ IU/100 μl, respectively. However, at a rFeIFN-ω concentration of 2 × 10^6^ IU/100 μl, the *BAX* expression level was slightly (1.12-fold) higher than in the control (Figure 2B).

The fold changes in the *BCL-2/BAX* transcript ratio were similar to the changes in the *BCL-2* expression level, where the ratio was 0.96- and 0.88-fold lower than in the control at rFeIFN-ω concentrations of 10^3^ and 10^5^ IU/100 μl, and were significantly higher (4.68-fold) at 10^4^ IU/100 μl (Figure 2C).

### The Relative mRNA Expression of the Drug Resistance Genes (*ABCB1* and *ABCG2*) in CTVT Cells Treated With rFeIFN-ω

**Figure 3:**
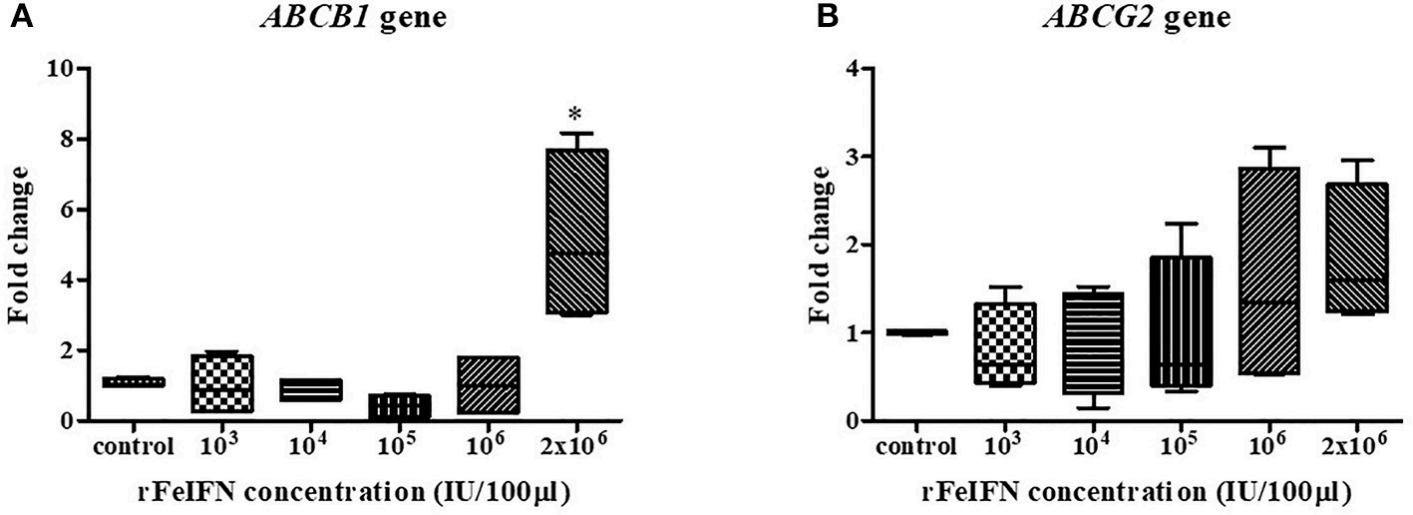
Box plot showed the relative mRNA expression of *ABCB1* **(A)** and *ABCG2* **(B)** gene of primary CTVT culture at 72-h after incubation with varied concentrations of rFeIFN-ω (^*^p < 0.05).

The relative mRNA expression of the *ABCB1* and *ABCG2* genes at 72 hpt was evaluated in the same manner as that for *BCL-2* and *BAX* genes above, with the results shown in Figure 3. The average relative transcript level of the *ABCB1* gene was decreased at low rFeIFN-ω concentrations by 0.98-, 0.88- and 0.43-fold at 10^3^, 10^4^, and 10^5^ IU/100 μl, respectively, compared to that of the control, and was unchanged at 10^6^ IU/100 μl and markedly (5.17-fold) and significantly increased at 2 × 10^6^ IU/100 μl (Figure 3A).

The relative expression level of *ABCG2* showed a similar pattern of change as that for *ABCB1*, with a 0.80-, 0.91- and 0.97-fold decrease compared to the control at rFeIFN-ω concentrations of 10^3^, 10^4^, and 10^5^ IU/100 μl, respectively, but was increased 1.58- and 1.84-fold at 10^6^ and 2 × 10^6^ IU/100 μl, respectively (Figure 3B). Thus, the primary CTVT culture contained a higher expression level of both *ABCB1* and *ABCG2* after treatment with rFeIFN-ω at 2 × 10^6^ IU/100 μl, although only the *ABCB1* relative transcript level was significantly higher than in the control.

## Discussion

Many studies have investigated the effect of rFeIFN-ω on tumor cells, where the ability of IFN to inhibit the proliferation of tumor cells was shown to be mediated by inhibition of cell cycle progression (10–12, 35). From our study, the viability of CTVT cells decreased when treated with increased concentrations of rFeIFN-ω. The reduction of CTVT proliferation is in agreement with previous reports showing the effect of IFN may cause by prolonging all phases of the cell cycle and upregulation of endogenous inhibitors of cyclin dependent kinases in a dose-dependent manner (35–37). In this study, the primary CTVT cells were sensitive, in terms of showing significant changes, to rFeIFN-ω at concentrations higher than 10^6^ IU/100 μl at 24 and 48 hpt, and at 10^5^ IU/100 μl at 72 hpt. Thus, the CTVT cells were sensitive to rFeIFN-ω not only in a dose-dependent manner but also with exposure time.

It was previously reported that rFeIFN-ω had a strong antiproliferative activity on feline cell lines, but a lower activity against a canine cell line where a higher rFeIFN-ω concentration was required to achieve an equal effect. These findings might imply a species-specific activity. Nevertheless, the dose-dependent property of rFeIFN-ω observed in this study was similar to previous reports (10–12), but we were unable to reveal the IC_50_ value (concentration of rFeIFN-ω required to inhibit 50% of cell growth).

Although the canine IFN-ω is unavailable, the efficacy of rFeIFN has still been detected in many other studies as well as in this one. The IFN-Is share the same signal pathway via an IFN alpha/beta receptor1 (IFNAR1)-IFNAR2 receptor, which binds all type I IFNs. A previous study of IFN-I in human tumors found that after IFN-I binding with its specific receptor at the cell membrane, the complex associates with JAK1-TYK2 and then IFN-I translocates to the nucleus and activates STAT. This signaling, which affects the cell proliferation through P21 and CDK1, results in an antiproliferative effect to the tumor cells (35, 36, 38, 39).

In addition, IFNs can induce apoptosis by the intrinsic and extrinsic pathways (35). The intrinsic apoptotic pathway has been associated with the function of the Bcl2 family (24). In this study, only at a rFeIFN-ω concentration of 10^4^ IU/100 μl was the relative mRNA expressions of *BCL-2* and *BAX* higher and lower, respectively, than in the control and the other rFeIFN-ω concentrations. Likewise, the *BCL-2/BAX* ratio showed a similar result to the relative mRNA expression of *BCL-2*. This increasing in *BCL-2* gene expression may be argued that CTVT survival cells after treated with rFeIFN-ω upregulated or preferentially expressed *BCL-2* gene. This result may state that there has been a positive selection of *BCL-2* expression cells *in vitro* under rFeIFN-ω treatment. This may then be the mechanism by which CTVT cells survive for a period of time after exposure to the rFeIFN-ω, as in this study. However, this result is still controversial, since this result was only found at a concentration of 10^4^ IU/100 μl.

Three ABC transporters (P-gp, MRP1 and BCRP) are the major drug transporters that facilitate the flux of molecules out of tumor cells. Normally, these transporters protect the cell by effluxing lethal or toxic substances out of the cells (26–28). At the highest rFeIFN-ω dose (2 × 10^6^ IU/100 μl) the relative mRNA expression of the *ABCB1* gene, which encodes P-gp was overexpressed compared to that in the control and the lower rFeIFN-ω concentration groups. However, the CTVT cells showed a response to rFeIFN-ω at only the highest concentration in this study. According to the result, the increasing significantly in *ABCB1* gene may be the result of the CTVT survival population after treatment. Those survival cells have possibility upregulated *ABCB1* and *ABCG2* gene that related to the drug efflux protein.

As previous research, CTVT cells share the same clonal origin; they have the same genetic feature and physiological behaviors (13–15). Following this study, the experimental design relies on a single primary culture of a single dog. However, we also recorded the chemotherapeutic outcome. This CTVT mass gave a complete regression after treated with vincristine sulfate. According to the previous research, Frampton et al. reported the molecular signature of regression of CTVT case (40). They also applied the biopsy from a dog as a model for the molecular signaling study of regression in CTVT after treated with vincristine sulfate. Thus, our *in vitro* result may apply as a model for precision therapy. However, the more informative and more valuable, the samples should cover all types of CTVT and drug-resistant cases in the future.

Based on the fact that the natural CTVT growth pattern is related to the host immune response. The previous study revealed that the combination of vincristine sulfate and interferon-α shorten the duration of treatment compared to vincristine alone. In addition, the results showed that the number of TILs in the combination treatment group was higher than the single chemotherapy (41). According to the result, vincristine sulfate may trigger the inflammation and activate the innate immune response, then induce the expression of interferon signaling genes and *CCL5* gene, which related with recruiting mononuclear cell during this early regression (40). So this combination treatment provides two aspects of tumor regression mechanism, firstly vincristine sulfate causes the inflammation triggering and inducing the interferon signaling expression then, induces the infiltration of lymphocytes and mononuclear cells. Secondly, the intratumoral interferon injection also enhances the ability of mononuclear cells and may cause the antiproliferative effect against the CTVT cells. From this point, the potential for *in vivo* interferon trial in further study for reducing the chemotherapeutic drug adverse effect could be accentuated.

This study was the first observation of an *in vitro* effect of rFeIFN-ω at various concentrations on a primary CTVT culture. According to the results, rFeIFN-ω may play an important role in the reduction of CTVT cell viability in a dose-dependent manner. In addition, the mRNA expression of *BCL-2* gene was quite higher in survival CTVT cells at a rFeIFN-ω concentration of 10^4^ IU/100 μl. Although, the higher rFeIFN-ω concentration leads to a greater inhibition of cell growth, the relative mRNA expression level of *ABCB1* transporter gene was upregulated to a greater extent. This might be the tumor cells responded to the lethal concentration and provide a feedback mechanism for survival. However, the antiproliferation ability of rFeIFN-ω and the detection of apoptotic cell were not performed in this study. Those two assays and the molecular signals to achieve the antiproliferative effect need to be clarified in the future studies. Moreover, this commercial feline-derived IFN might encourage performing experimental *in vitro* study in CTVT resistant case and eventually *in vivo* trials to evaluate the efficacy of this treatment in CTVT cases.

## Ethics Statement

This study was approved by the Chulalongkorn University Animal Care and Use Committee (No. 133100077).

## Author Contributions

CS participated in sample collection, experiments, and writing the manuscript. SiT contributed for experimental design. AR was responsible for experimental design and supervising of clinical studies with dogs. SoT participated in designing experiments, compiling results, and revised the manuscript.

### Conflict of Interest Statement

The authors declare that the research was conducted in the absence of any commercial or financial relationships that could be construed as a potential conflict of interest.

## Supplementary Material

Supplementary Figure 1 Cauliflower-like mass located at the penis. Before treatment, this active mass was reddish, oozing and easy to bleed.
