# Supplementary figures and images for: In vitro Effect of Recombinant Feline Interferon-Ω (rFeIFN-Ω) on the Primary CanineTransmissible Venereal Tumor Culture
Source: Front Vet Sci. 2019 Apr 9;6:104. doi: 10.3389/fvets.2019.00104 (PMC6467026; doi:10.3389/fvets.2019.00104)

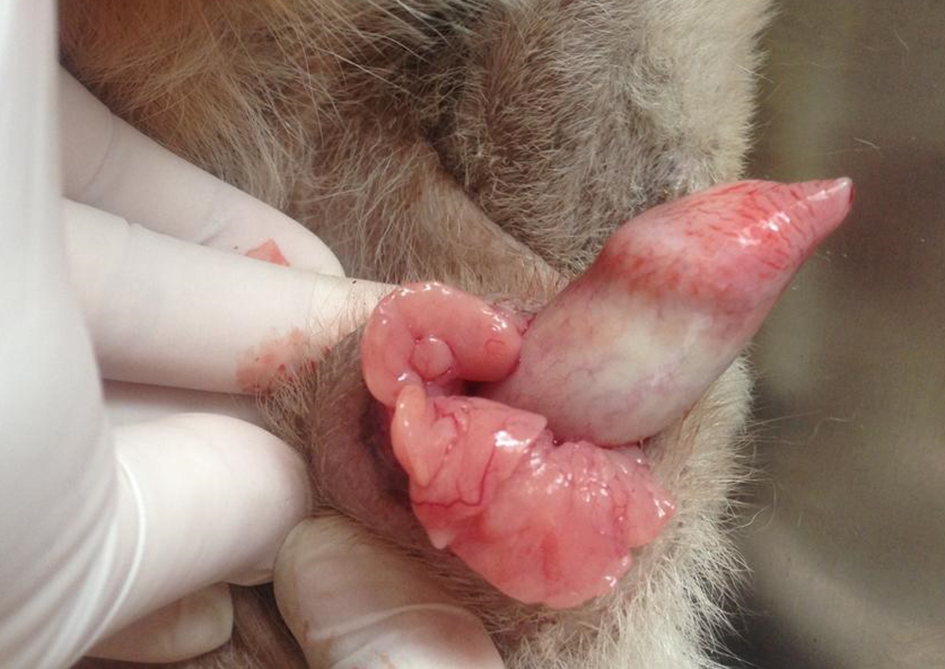

Supplement: Supplementary Figure 1 — Cauliflower-like mass located at the penis. Before treatment, this active mass was reddish, oozing and easy to bleed. [file Image_1.jpg]
